# Geographical distribution and evolutionary dynamics of H4Nx avian influenza viruses

**DOI:** 10.3389/fmicb.2024.1505203

**Published:** 2025-01-07

**Authors:** Ye Ge, Jing Liu, Yuanguo Li, Peng Peng, Yan Zhou, Jiantao Yu, Miaotong Huo, Xiaodong Liang, Yuwei Gao, Qiucheng Yao

**Affiliations:** ^1^Guangdong Ocean University, Zhanjiang, China; ^2^Changchun Veterinary Research Institute, Chinese Academy of Agricultural Sciences, Changchun, China; ^3^Center for Biological Disaster Prevention and Control, National Forestry and Grassland, Shenyang, China; ^4^Forestry Administration of Guangdong Province, Guangzhou, China

**Keywords:** avian influenza virus, H4Nx, genetics, viral evolution, phylogeographic analysis

## Abstract

H4Nx avian influenza viruses (AIVs) have been isolated from wild birds and poultry and can also cross the species barrier to infect mammals (pigs and muskrats). The widespread presence of these viruses in wild birds and poultry and their ability to be transmitted interspecies make them an undeniable hazard to the poultry farming industry. In the present study, we collected fecal and swab samples from wild birds and poultry in Guangdong Province from January 2019 to March 2024, and various subtypes of AIVs were isolated, including 19 strains of H4 subtype AIVs. Further analysis was conducted on the internal genes of the 19 strains. These strains clustered together with high homology to highly pathogenic avian influenza virus (HPAIV), suggesting that H4Nx AIV may be reassorted from HPAIV. Two H4N8 strains are phylogenetically related to the porcine H4N8 AIV. Molecular characterization revealed that all viruses in this study were less pathogenic but had potential mammalian-adapted mutations. The transmission dynamics of H4Nx AIVs revealed that Europe and Asia, especially the Netherlands and Bangladesh, may be the centers of transmission. This may be linked to the migration of wild birds. The high migration rates from Russia to the Netherlands and from Russia to Bangladesh may also play a role. Therefore, continuous and systematic monitoring of wild birds to clarify the spatial and temporal distribution and prevalence of influenza viruses in wild birds is significant for early warning of avian influenza outbreaks in poultry and for risk assessment for public health and safety.

## Introduction

Avian influenza virus (AIV) is a single-stranded, negative-stranded, segmented RNA virus consisting of eight gene segments belonging to the genus influenza A of the family Orthomyxoviridae (Krammer et al., 2018). Based on differences in the viral surface antigenic proteins hemagglutinin (HA) and neuraminidase (NA), the AIV has been categorized into 18 HA subtypes and 11 NA subtypes, all of which can be isolated from wild birds, except subtypes H17N10 and H18N11 (Tong et al., 2013). Moreover, AIV has a wide host range, infecting birds, poultry, mammals, and even humans, including aquatic birds, especially waterfowls, gulls, and shorebirds. Aquatic birds are considered the natural hosts of the AIV, from which all subtypes of the influenza virus can be isolated (Araujo et al., 2018; Lycett et al., 2019).

In 1956, H4 AIV was isolated for the first time from a duck in Czechoslovakia (Donis et al., 1989), and subsequently spread to Asia, Europe, and North America. Between 2019 and 2021, a total of 4,451 wild bird samples were collected in Shanghai, and 45 strains of H4 subtypes were identified, proving that migratory activities play an important role in the transmission of AIVs between wild birds and poultry (Xu et al., 2023). Moreover, H4 AIV can be transmitted from wild birds to poultry and replicate efficiently in the poultry respiratory tract (Bergervoet et al., 2019; Root et al., 2022). The H4 subtype AIV is transmitted between wild birds and poultry and can also cross species barriers to infect mammals. The first isolation of the H4N6 subtype of AIV in a pig in Canada in 2000 (Karasin et al., 2000) and subsequent isolation in pigs in China (Hu et al., 2012) and the United States (Abente et al., 2017). In October 2011, a pig was reported to be infected with the avian-origin H4N8 virus, which had the highest homology of the HA gene to avian-origin H4N6 isolated from Japan and the highest homology of the NA gene to chicken-origin H3N8 virus from Guangxi (Su et al., 2012). This suggests that the H4N8 virus may have been produced by recombination between chickens and avians. In 2012, a strain of the H4N6 subtype AIV was isolated from seals in the Caspian Sea (Gulyaeva et al., 2018). It has been shown that the H4 AIVs pose a potential threat to public health, replicate in mice without prior adaptation, bind to the human-type receptor (α-2,6-SA), and can be transmitted between guinea pigs by direct contact, and some viruses can be transmitted through respiratory droplets (Liang et al., 2016). Moreover, seroepidemiological surveys have found that H4 AIV can infect poultry farmers in Lebanon (Kayali et al., 2011) and the United States of America (Kayali et al., 2010). There is growing evidence that H4 AIV can cross the species barrier and infect both mammals and humans. Therefore, enhanced ongoing surveillance of H4 AIV is necessary.

Nine global migratory routes are known for birds (Olsen et al., 2006). Guangdong Province is located under the East Asia-Australia migration route, which is an important area for migratory birds to meet and stop. Moreover, Guangdong Province has a well-developed water network and is dominated by waterfowl farming, which often congregates near rivers, reservoirs, and wetlands, facilitating contact between wild birds and poultry and providing conditions for long-distance transmission of AIVs. In this study, virus isolation was performed on poultry and wild bird samples collected during 2019–2024 in Guangdong Province. All H4 AIV isolates were comprehensively and systematically analyzed for epidemiological investigation, phylogenetic analysis, phylogeographic analysis, and molecular characterization to elucidate the transmission dynamics and evolutionary patterns of H4 AIV.

## Materials and methods

### Eggs

Specific pathogen-free (SPF) embryonated chicken eggs were purchased from Beijing Vital River Laboratory Animal Technology Company of Boehringer Ingelheim (China) and incubated to 9–11 days of age for virus isolation at 37°C and 85% humidity.

### Sample collection and virus isolation

A total of 8,492 fecal samples of wild birds and oropharyngeal and cloacal specimen samples of poultry were collected from 20 reserves in Guangdong Province. These samples were preserved in phosphate-buffered saline buffer containing antibiotics and glycerol, put into a thermostat set at 4°C for low-temperature preservation, and transported to the laboratory in time and transferred to a −80°C refrigerator for preservation for subsequent experiments.

The collected samples were mixed by shaking and freeze-centrifuged at 5,000 rpm for 10 min and then inoculated into SPF chicken embryos through the allantoic cavity, blindly transmitted for two generations, and cultured in an incubator set at 37°C for 72 h, with the embryos photographed every 24 h. After 72 h, all chicken embryos were placed in a refrigerator at 4°C overnight. The hemagglutination of the allantoic fluid of chicken embryos was detected using 1% chicken red blood cells. Erythrocytes of SPF chickens were obtained from Guangdong Xinxing Dahuanong Poultry Egg Company. The allantoic fluid of hemagglutination-positive chicken embryos was collected for the next step of identification.

### Whole-genome sequencing

RNA was extracted from allantoic fluid using a nucleic acid extraction kit and then reverse transcribed using a reverse transcription kit. According to national standards (GB/T 18936-2020), reverse transcription-polymerase chain reaction (RT-PCR) was performed using identification primers (M-229U: TTCTAACCGAGGTCGAAAC; M-229L: AAGCGTCTACGCTGCAGTCC). Expected sizes of the amplified fragments were considered positive.

The whole-genome sequencing (WGS) of AIV was determined using second-generation sequencing. The sequencing data were combined using the SeqMan package of DNAstar (11.1.0) software. WGS of all H4 subtype AIVs up to March 2024 were downloaded from the National Center for Biotechnology Information (NCBI) and Global Initiative on Sharing All Influenza Data (GISAID) databases. Sequences were imported into MEGA 7.0 software, and duplicate sequences were deleted. Information on sampling time, sampling location, and host was summarized to analyze the prevalence of AIV subtype H4.

### Evolutionary analysis of genetics

The spliced sequences were analyzed using the NCBI Basic Local Alignment Search Tool (BLAST), the top 100 sequences with the highest homology and typical sequences were downloaded and analyzed, and duplicated sequences were deleted. Sequences with complete coding regions were selected, and a phylogenetic analysis of six internal genes (M, NS, NP, PA, PB1, and PB2) was performed using the maximum likelihood method (ML) using the MEGA 7.0 software package (1,000 bootstrap replicates).

WGS of the H4 subtype AIV was downloaded from the NCBI and GISAID databases. For efficient analysis, sequences with 99.9% homology were deleted using BioAider V 1.527 software, and those from different times, hosts, and collection sites were retained. The common ancestry (tMRCA) and evolutionary rate of the H4N6 subtype avian were estimated using the BEAST software package. Sequence alignment was performed using MAFFT. The sequences were trimmed to preserve the coding regions, and the best nucleotide substitution model was selected using ModelFinder. ML trees were plotted using IQ-tree, with the bootstrap set to 1,000. The sequences were checked using the TempEst software to determine whether they had sufficient temporal signals for phylogenetic molecular clock analysis. The XML file was formulated using the BEAUti software by selecting two combinations of strict and relaxed molecular species with constant size, exponential growth, and Bayesian skyline tree models. The Bayesian Markov Chain Monte Carlo (MCMC) chain length was set to 5 million generations and sampled every 5,000 steps. Convergence of the results was achieved by running the “log” file through Tracer evaluation with an effective sample size (ESS) value of >200. Finally, the converged model was selected and repeated three times to plot the best evolutionary tree. Eventually, the top 10% of the sampling results were removed (bum-in) using TreeAnnotator, and the MCMC tree was combined using LogCombiner to generate the maximum clade confidence (MCC) tree. The tree was identified using Treefile software.

### Evolutionary dynamics analysis

To evaluate the effective population sizes of AIVs of subtypes H4N6, H4N2, and H4N8, skyline plots were used to infer the statistical population histories of the H4N6, H4N2, and H4N8 viruses. TempEst software was used to evaluate the temporal signals of the IQ-tree and to estimate the relative genetic diversity of the HA genes of the H4N6, H4N2, and H4N8 viruses using BEAST analysis.

### Spatio-temporal dynamics analysis

Spatial and temporal migration paths of the H4Nx virus based on Bayesian phylogeography of the HA gene. The HA sequences of H4Nx AIV were downloaded from the NCBI database, duplicates and missing sequences were removed, and sequences from different times and sampling locations were selected for analysis to draw the IQ-tree. The root-to-tip genetic distance of the IQ-tree was regressed using TempEst to determine the sampling date. A strict molecular clock model is used. Additionally, phylogeographic analysis was performed using a Bayesian Stochastic Search for Variable Selection (BSSVS) model. For each independent dataset, 5,000,000,000 steps were used, and sampled every 5,000 steps. MCMC was combined using LogCombiner.

Subsequently, we calculated the Bayes factor (BF) using SpreaD3 v0.9.6 software to assess support for significant inter-individual differences between geographic locations: BF >100 indicates extremely strong statistical support, 30–100 indicates very strong statistical support, 10–30 indicates strong statistical support, 3–10 indicates moderate statistical support, and BF <3 indicates weak statistical support.

### Evolutionary rate analysis

The full gene sequences of H4N2, H4N6, and H4N8 were downloaded from the NCBI database. Duplicates and poor sequencing quality sequences were removed, and selective pressures were calculated using DNA Sequence Polymorphism v6.12.03 software. Selection pressure was evaluated based on d*N*/d*S*, with d*N*/d*S* < 1 indicating negative selection, d*N*/d*S* = 1 representing neutral selection, and d*N*/d*S* > 1 suggestive of positive selection.

### Molecular character analysis

The percent identity of the H4Nx virus was calculated based on consensus nucleotide sequences. Single-nucleotide polymorphisms and amino acid substitutions resulting from nucleotide polymorphisms have been analyzed in H4Nx viral populations isolated from different hosts. Key amino acid sites in the HA, NA, PB2, PA, and M genes were analyzed to understand the potential biological features of H4Nx viruses, including their receptor binding preference, replication, virulence, and antiviral drug susceptibility.

### Statistical analysis

Base composition data were graphically plotted using GraphPad Prism (version 10.0.0) and modified using Adobe Illustrator 27.5.

## Results

### Isolation and characterization of H4 subtype AIV

Twelve subtypes of AIV (H3N6, H3N8, H4N6, H4N2, H4N8, H6N2, H6N6, H6N8, H7N3, H8N4, H10N5, and H10N7) were identified, with 19 strains of the H4 subtype of AIV, with a 0.22% positivity rate. H4 AIVs contained three combinations: H4N2, H4N6, and H4N8. Among these H4 AIVs, H4N2, H4N6 and H4N8 accounted for 10.53% (2/19), 78.94% (15/19) and 10.53% (2/19), respectively. One strain of H4N8 originated from ducks, and the rest originated from wild birds, with five viral strains of H4N6 isolated from green-winged ducks. Specific information on these 19 viral strains is presented in Table 1.

**Table 1:** Information of the 19 H4 subtype avian influenza viruses isolated in wild birds in China.

Name	Abbreviation	Sampling time	Host	Subtype
A/common teal/Guangdong/177/2023	177	2023.11.1	Common teal	H4N6
A/common teal/Guangdong/74/2023	74	2023.11.1	Common teal	H4N6
A/common teal/Guangdong/60/2023	60	2023.11.1	Common teal	H4N6
A/common teal/Guangdong/41/2023	41	2023.11.1	Common teal	H4N6
A/common teal/Guangdong/352/2023	352	2023.11.1	Common teal	H4N6
A/common teal/Guangdong/289/2023	289	2023.11.1	Common teal	H4N2
A/wild duck/Guangdong/ZJ1570/2023	ZJ1570	2023.12.9	Wild duck	H4N2
A/wild duck/Guangdong/ZJ1634/2023	ZJ1634	2023.12.9	Wild duck	H4N6
A/wild duck/Guangdong/ZJ1586/2023	ZJ1586	2023.12.9	Wild duck	H4N6
A/wild duck/Guangdong/ZJ1686/2023	ZJ1686	2023.12.9	Wild duck	H4N6
A/wild duck/Guangdong/ZJ1534/2023	ZJ1534	2023.12.9	Wild duck	H4N6
A/wild duck/Guangdong/ZJ1566/2023	ZJ1566	2023.12.9	Wild duck	H4N6
A/wild duck/Guangdong/ZJ1498/2023	ZJ1498	2023.12.9	Wild duck	H4N6
A/wild duck/Guangdong/ZJ1506/2023	ZJ1506	2023.12.9	Wild duck	H4N6
A/wild duck/Guangdong/ZJ1674/2023	ZJ1674	2023.12.9	Wild duck	H4N6
A/wild bird/Guangdong/ZJ476/2022	ZJ476	2022.11.20	Wild bird	H4N6
A/wild duck/Guangdong/ZJ1028/2023	ZJ1028	2023.1.6	Wild duck	H4N6
A/wild bird/Guangdong/M50/2021	M50	2021.11.3	Wild bird	H4N8
A/duck/Guangdong/DK2/2019	DK2	2019.3.28	Duck	H4N8

### World prevalence of subtype H4 subtype AIVs

**Figure 1:**
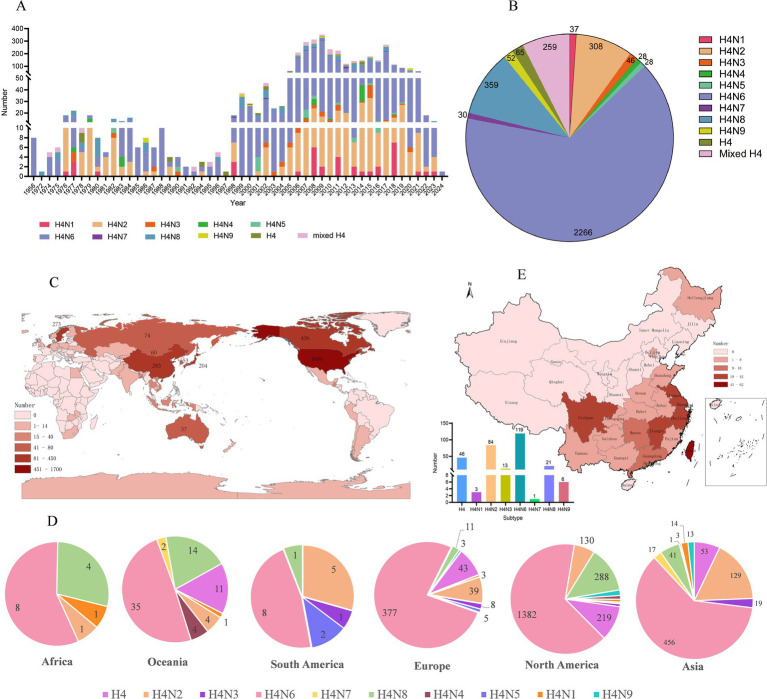
Epidemiologic investigation of H4 subtype AIVs worldwide. **(A)** Number and subtype distribution of H4 viruses in the database from 1956 to March 2024. Different colors represent different subtypes, the *x*-axis indicates the time of virus isolation, and the *y*-axis indicates the number of viruses isolated. **(B)** Distribution of the number of different subtypes of H4Nx AIVs. **(C)** Global distribution of H4 subtype AIVs, with darker colors representing higher numbers. **(D)** Subtype distributions were quantified separately for Asia, Africa, Europe, North America, Antarctica, and Oceania, with only one strain of H4N7 noted in Antarctica. **(E)** Number, geographic location, and subtype distribution of H4 subtype AIVs in China from 1956 to March 2024.

To better characterize the global epidemiology of H4 AIV, we systematically analyzed their the prevalence of H4 AIV according to time, place, and host distribution. All H4 AIV sequences in the NCBI and GISAID databases as of March 2024 were downloaded, yielding 3,479 AIVs of the H4 subtype. The analysis showed that the highest number of H4 subtype AIVs was detected during the period 2005–2021 compared to other years, with the highest number of strains (352) detected in 2009. Overall, the number of H4 AIV isolates increased annually (Figure 1A). Analysis of the subtypes spread of H4 AIV revealed that the most predominant subtype was H4N6 (2,267 strains), which accounted for 65.13% (2,266/3,479) of all subtypes and was isolated in all years except 1972 and 1997. This was followed by H4N2 (308 strains) and H4N8 (359 strains); H4N4 (28 strains) and H4N5 (28 strains) were the least frequently isolated (Figures 1A,B). The results showed that the H4N6 subtype was the dominant strain of the H4 subtype AIV.

According to the statistical analysis of the global data of H4 subtype AIV downloaded as of March 2024, the results showed that H4 subtype AIV was distributed in numerous regions, among which the United States had the most isolated AIV, accounting for 48.40% (1,684/3,479) of the world, followed by Canada, China, Sweden, and Japan with more isolations, accounting for 12.53% (436/3,479), 8.42% (293/3,479), 7.85% (273/3,479), and 5.86% (204/3,479), respectively (Figure 1C). H4Nx has nine subtypes, of which Europe, Asia, and North America are the most abundant in terms of subtype variety, with H4N6 and H4N2 being predominantly prevalent in Asia and Europe, and H6N6 and H4N8 subtypes being predominant in North America. H4N7 subtypes have only been isolated in Asia, North America, and Antarctica. H4N6 emerged as the most prevalent subtype on all continents (Figure 1D).

A total of 293 AIV strains of the H4Nx subtype were identified in China, primarily concentrated in the southern region, with more isolates from Hong Kong (62 strains), Jiangxi (42 strains), Sichuan (33 strains), Shanghai (32 strains), Jiangsu (25 strains), and Zhejiang (25 strains). The subtypes isolated from China were more abundant, with H4N6 and H4N2 being the most prevalent (Figure 1E).

### Host distribution of the H4 subtype AIVs

**Figure 2:**
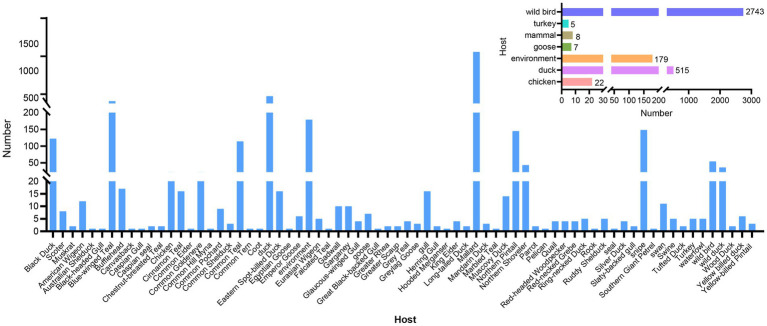
Statistics on host distribution of H4 subtype AIVs. The main image reflects the detailed host source of the virus, and the secondary image reflects the broad categories of hosts from which the virus originated.

Additionally, H4 subtype AIVs showed a wide host distribution and could infect 72 different hosts. H4 subtype AIVs primarily infected wild birds, accounting for 78.73% (2,743/3,479) of the total number of hosts recorded in the database. The Common Mallard was the primary host of wild birds, accounting for 49.25% (1,351/2,743) of the wild bird population, followed by the blue-winged teal. The isolation rate from blue-winged teal was 16.11% (442/2,743) in the wild bird population. H4 subtype AIVs surveillance in poultry is at the second level. The H4Nx subtype AIVs can also infect mammals, with an isolation rate of 0.23% (8/3,479) in pigs, muskrats, and seals. The isolation rates from H4Nx in ducks, geese, chickens, environment, and turkeys were 14.80% (515/3,479), 0.20% (7/3,479), 0.63% (22/3,479), 5.15% (179/3,479), and 0.14% (5/3,479), respectively (Figure 2). Statistically, H4 subtype AIVs were primarily isolated from waterfowl, such as ducks, geese, and wild ducks, which are the primary hosts of H4 viruses.

### Phylogenetic analysis

**Figure 3:**
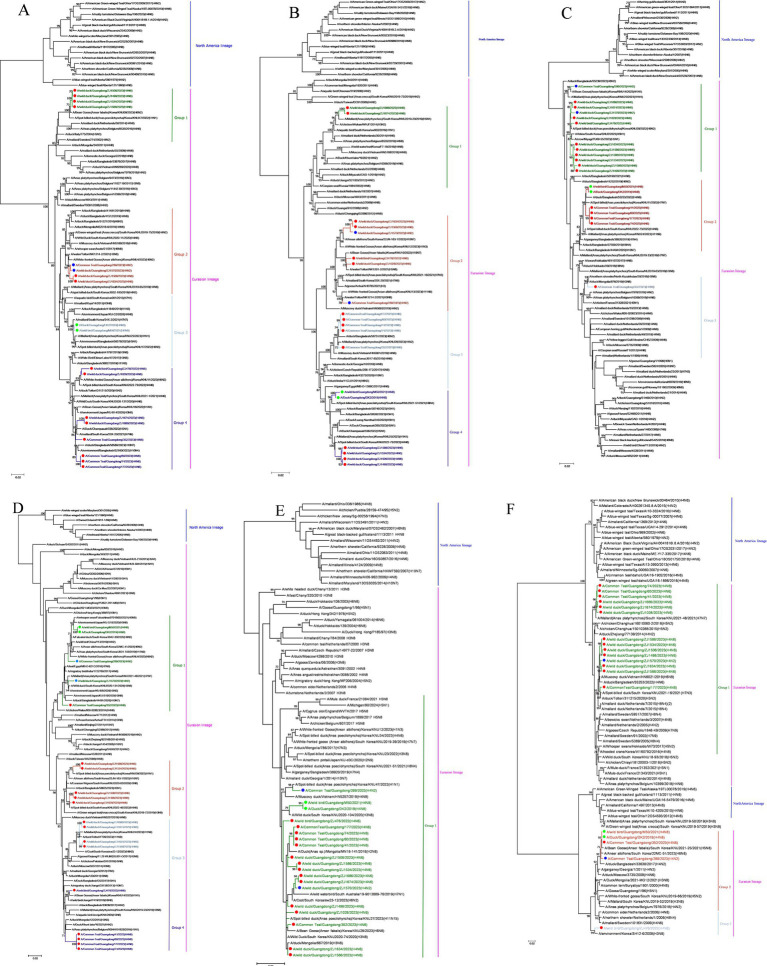
Phylogenetic tree of the six internal genes of isolated viral strains. The six genes are **(A)** PB2, **(B)** PB1, **(C)** PA, **(D)** NP, **(E)** M, and **(F)** NS. The tree was generated using the maximum likelihood method and bootstrapped with 1,000 replicates using the MEGA 7.0 software. Isolates are indicated with colored circular symbols (red, H4N6; blue, H4N2; green, H4N8).

The 19 viruses isolated in this study were categorized into 8 genotypes (>96% nucleotide identity in one group) based on the phylogenetic diversity of each gene fragment and the group of each gene fragment (Supplementary Figure S1). Six internal genes of the H4Nx subtype AIVs were phylogenetically analyzed, and a phylogenetic tree was constructed. The internal genes of the 19 AIV strains isolated in this study belonged to the Eurasian lineage (Figure 3). The PB2 gene showed 93.0–100% nucleotide homology and was classified into four groups. Of particular interest were two strains of H4N8 subtype AIVs (DK2 and M50) that clustered in the same branch as highly pathogenic AIVs [A/environment/Bangladesh/58578/2023 (H5N1)]. H4N6 in Group 4 was on the same branch as H5N1 HPAIV (A/Duck/Champasak/556/2022) of duck origin (Figure 3A). The PB1 gene with 92.3–99.9% nucleotide homology, was classified into four groups, in which H4N2 AIVs were found in Group 2, H4N8 AIVs in Group 4, and H4N6 AIVs in all four groups. Notably, H4N8 and H4N6 in Group 4 clustered with HPAIV H5N1 (Figure 3B). The PA gene showed 95.8–100% nucleotide homology and was classified into three groups. The AIVs in Group 1 were in the same group as HPAIV H5N1 and were highly homologous. H4N8 and H4N6 in group 2 have high nucleotide homology with H7N7, H11N9, H7N4, H8N4 and H7N4. Group 3 contained only strain 352 (H4N6). However, it was also in the same group as HPAIV H5N1 with a high degree of homology (Figure 3C). The NP genes were 92.7–100% homozygous and were classified into four groups. H4N8 and H4N2 were in Group 1, clustered with highly pathogenic H5N8 subtype AIVs [A/whooper swan/Fukushima/0701B002/2021 (H5N8) and A/environment/Japan/KU-G18/2020 (H5N8)] clustered together. The H4N6 subtype AIVs in groups 2, 3, and 4 clustered with the HPAI viruses (Figure 3D). The M gene has 97.8–100% nucleotide homology and high homology, which was divided into one group and was in the same group as HPAI viruses H5N1 and H5N8, with >96% homology (Figure 3E). The NS gene has 71–100% nucleotide homology and is classified into three groups, all belonging to the Eurasian branch but also in the same large branch as the North American branch. Strains M50, DK2, 289, and 352 were in the same branch as the A/Goose/Guangdong/1/96 (H5N1) strain with 95.7% homology (Figure 3F).

**Figure 4:**
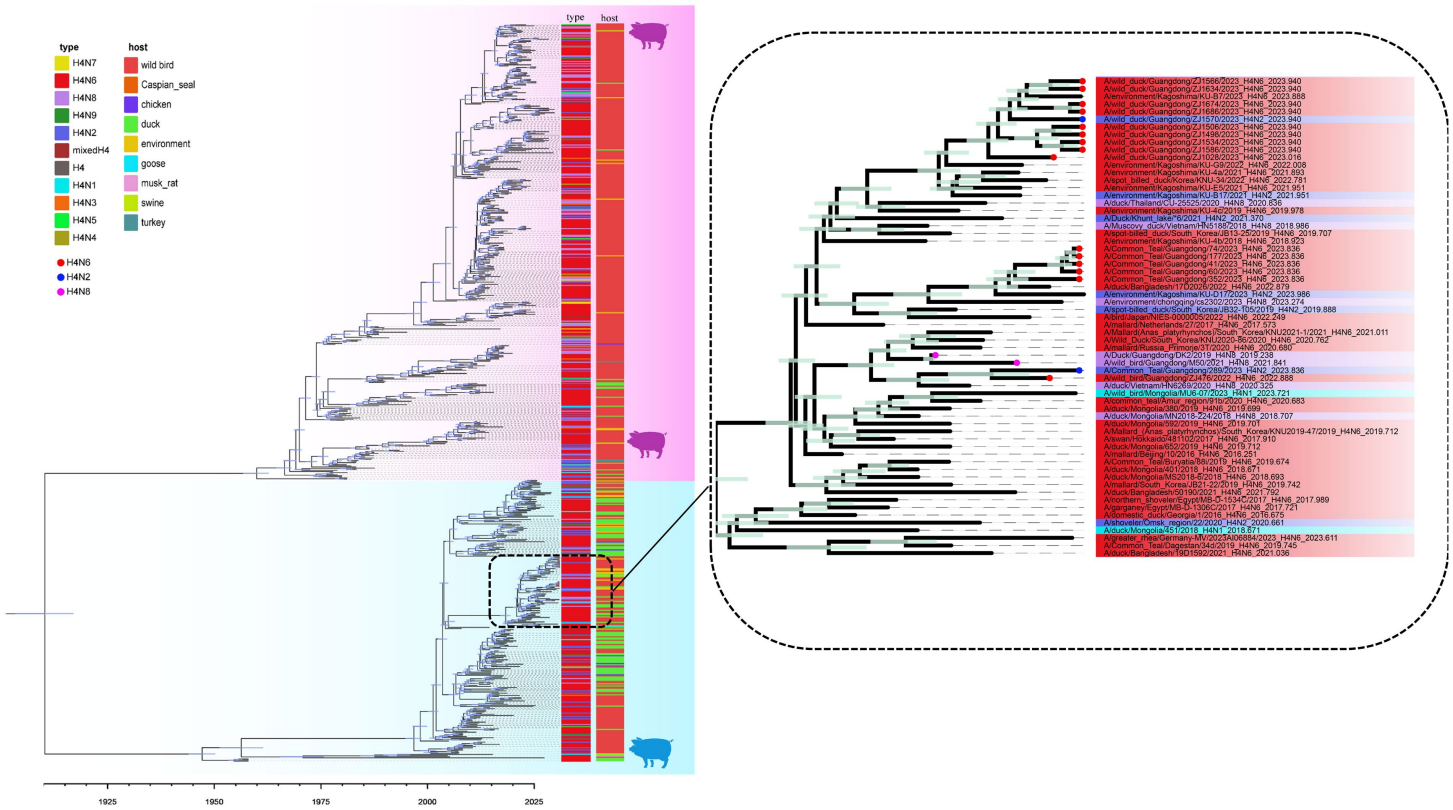
Time-scaled evolution of the HA gene of H4Nx viruses. Different subtypes and hosts are indicated with different colored squares. The strains isolated in this study are labeled with different colored circles: H4N6, red; H4N2, blue; and H4N8, purple. Shaded bars represent 95% highest probability distribution for the age of each node.

**Figure 5:**
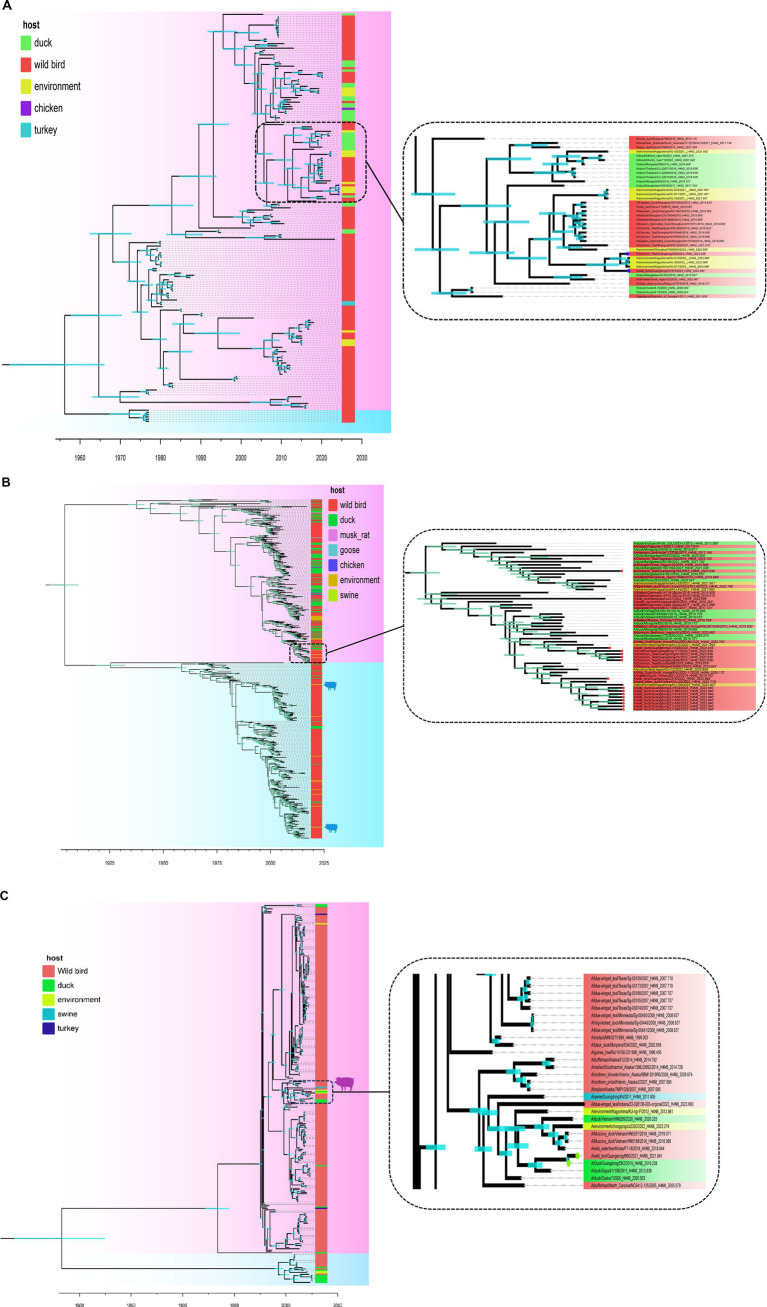
Time-scaled evolution of the NA gene of H4Nx viruses. **(A)** H4N2, **(B)** H4N6, **(C)** H4N8. The colors of squares represent different hosts. In this study, H4N2 strains are represented by blue circles, H4N6 strains are represented by red circles, and H4N8 strains are represented by green circles. Shaded bars represent 95% highest probability distribution for the age of each node.

A MCC was constructed for the H4Nx AIV surface genes, showing two major branches, the Eurasian and North American branches, and all H4Nx viruses isolated in this study belong to the Eurasian branch. In the HA-MCC tree, the branch host type where the viruses isolated in this experiment were located was rich and was also the primary distribution area of the domestic duck-derived viruses (Figure 4). Phylogenetic analyses of N2 (Figure 5A), N6 (Figure 5B), and N8 (Figure 5C) revealed that the isolated viruses were in the same branch as those of poultry origin and that there were multiple host species. The NA gene of the H4N8 subtype clustered in the same branch as the porcine-derived AIV (A/swine/Guangdong/K4/2011).

### Evolutionary dynamics of H4 subtype viruses

To further analyze the dynamic evolution of the H4Nx subtype AIV, the temporal structure was revealed by root-to-tip regression for HA (*n* = 662, correlation coefficient = 0.9152, *R*^2^ = 0.8376), N2 (*n* = 182, correlation coefficient = 0.9356, *R*^2^ = 0.8753), N6 (*n* = 588, correlation coefficient = 0.9224, *R*^2^ = 0.8509), and N8 (*n* = 282, correlation coefficient = 0.8452, *R*^2^ = 0.7143) aspects of clock structure. The times of origin of H4, N2, N8, and N6 were estimated from the 95% highest probability density (HPD) as follows: HA (December 1904–September 1917); N2 (September 1941–June 1965); N6 (June 1897–April 1911); and N8 (October 1739–February 1825). These findings suggest that the origin of N8 (August 1783) precedes those of N6 (June 1904) and N2 (May 1954) (Table 2).

**Table 2:** Evolutionary rates of surface genes and the timing of recent common ancestry.

Segment	Correlation coefficient	*R* ^2^	Slope (rate)	Best-fit model	Median TMRCA	Low 95% HPD	Upper 95% HPD
H4	0.9152	0.8376	1.65 × 10^−3^	GTR-F-R5	July 1911	December 1904	September 1917
N2	0.9356	0.8753	1.79 × 10^−3^	GTR-F-I-G4	May 1954	September 1941	June 1965
N6	0.9224	0.8509	1.89 × 10^−3^	GTR-F-R5	June 1904	June 1897	April 1911
N8	0.8452	0.7143	1.27 × 10^−3^	GTR-F-I-G4	August 1783	October 1739	February 1825

**Figure 6:**
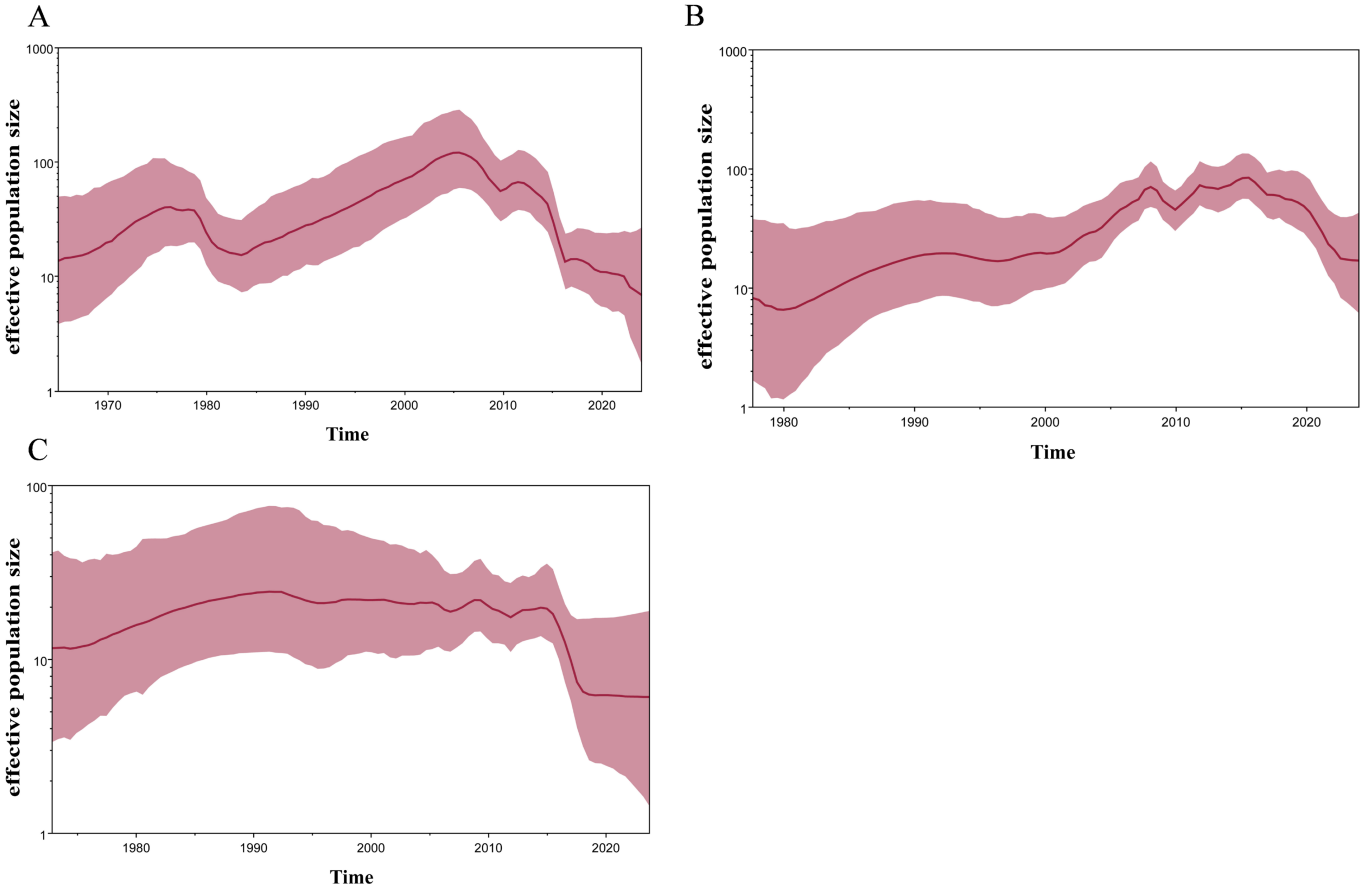
GMRF Bayesian Skyride analysis of HA genes **(A)** H4N2, **(B)** H4N6, and **(C)** H4N8 showing changes in the effective population size over time. The solid red line indicates median value, and the shaded red area represents 95% highest posterior density of genetic diversity estimates.

To analyze the genetic diversity of the different subtypes of H4 viruses, we inferred the demographic histories of H4N6, H4N2, and H4N8 viruses using GMRF Bayesian Skyride plots. The effective population size of H4N2 decreased in 1975 and increased continuously during 1985–2006, reaching a maximum value. Subsequently, the effective population size exhibited a fluctuating downward trend (Figure 6A). H4N6 showed a flat increase in the effective population size until 2009, with a peak increase in role in 2009, followed by a decline, and then another increase during 2010–2017, followed by a declining trend after 2017 (Figure 6B). H4N8 showed less population fluctuations until 2016 and leveled off after 2019 (Figure 6C).

### Spread of H4 subtype influenza viruses in China

**Figure 7:**
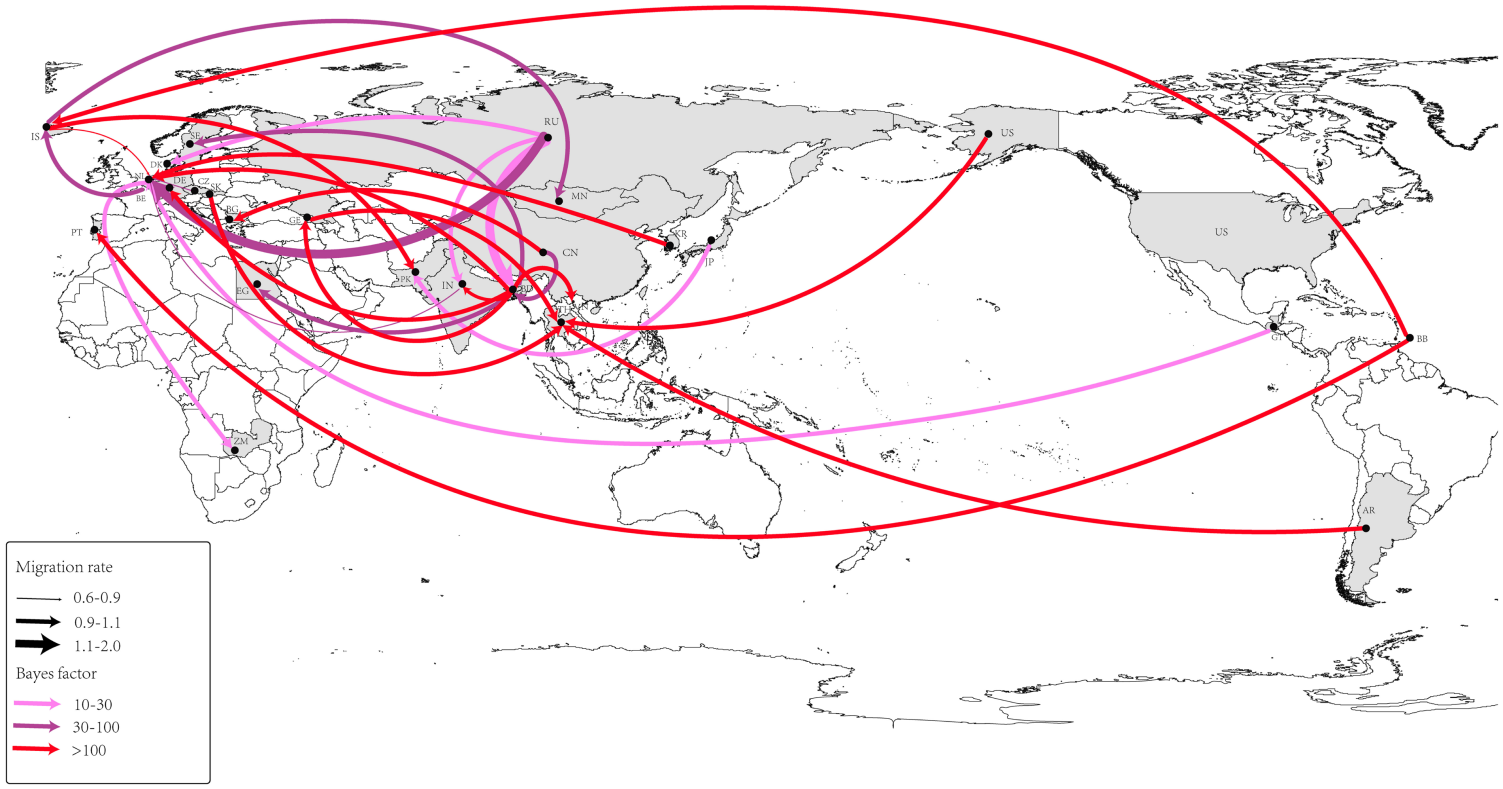
Spatio-temporal spread of H4Nx AIVs was determined using Bayesian geographic inference of the H4Nx AIV HA gene sequence. Curves show the spread paths of H4NX viruses with BF >3 statistical support. Curve widths indicate migration rate values and curve colors indicate the corresponding Bayes factor values for each migration rate.

**Figure 8:**
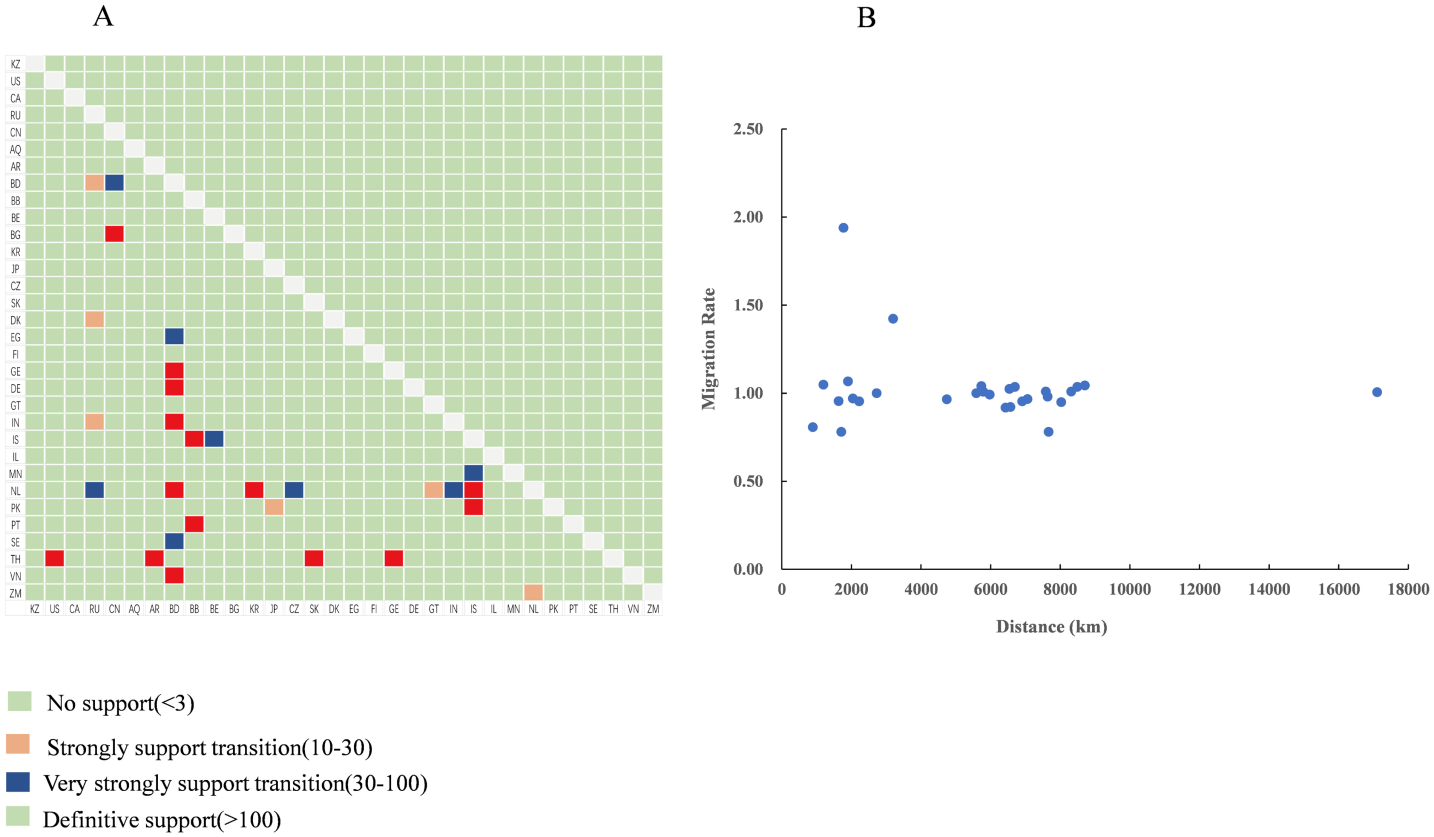
Transmission dynamics of the H4Nx virus. **(A)** Bayes factor support levels for each transmission route of the H4Nx AIV, with the *x*-axis indicating the origin location and the *y*-axis indicating the destination location. **(B)** Relationships among distances between countries and viral migration rates (BF >3), with no significant correlation.

Using the BSSVS approach, we constructed a worldwide spatial transmission network of H4Nx AIVs, selecting data with a BF of >3 and posterior probability (pp) of >0.5 for analysis. We screened 27 discrete sampling points with a BF >3 and identified 15 significant transmission paths (Table 3). The results of our study indicate that Europe and Asia are the major centers of transmission of H4Nx AIVs, especially the countries of the Netherlands (NL) and Bangladesh (BD), with the Netherlands having virus transmission with eight countries, including Zambia (BF = 19), India (BF = 69), Iceland (BF = 65,918), the Czech Republic (BF = 45), Guatemala (BF = 28), Bangladesh (BF = 14,637), South Korea (BF = 1,066), and Russia (BF = 44). There are nine countries in Bangladesh with transmission links: Vietnam (BF = 131,851), Georgia (BF = 131,851), Germany (BF = 131,851), the Netherlands (BF = 14,637), Sweden (BF = 54), India (BF = 4,381), Egypt (BF = 95), China (BF = 72), and Russia (BF = 22). A strong statistical support was observed for the migration of AIVs from China to Bulgaria (BF = 577) (Figure 7). Additionally, no significant correlation was found between the viral migration rate and the distance between the sampling locations (Figure 8). However, viral migration rates were found among countries that were closer together, such as Russia, Bangladesh (migration rate = 1.42), and the Netherlands (migration rate = 1.94). The United States to Thailand and Argentina to Thailand were further away and exhibited moderate migration rates.

**Table 3:** Statistically supported migration rates of H4Nx influenza viruses estimated from HA gene sequences.

From	To	Bayes factor	Posterior probability	Migration rate
Russia (RU)	India (IN)	16	0.52	1.00
Netherlands (NL)	Zambia (ZM)	19	0.56	0.98
Russia (RU)	Bangladesh (BD)	22	0.60	1.42
Russia (RU)	Denmark (DK)	23	0.61	0.96
Guatemala (GT)	Netherlands (NL)	28	0.66	1.01
Japan (JP)	Pakistan (PK)	29	0.67	0.92
Iceland (IS)	Mongolia (MN)	34	0.70	1.01
Belgium (BE)	Iceland (IS)	38	0.72	0.97
Russia (RU)	Netherlands (NL)	44	0.75	1.94
Czech Republic (CZ)	Netherlands (NL)	45	0.75	0.81
Bangladesh (BD)	Sweden (SE)	54	0.79	1.04
India (IN)	Netherlands (NL)	69	0.83	0.78
China (CN)	Bangladesh (BD)	72	0.83	1.07
Bangladesh (BD)	Egypt (EG)	95	0.87	0.99
Barbados (BB)	Iceland (IS)	110	0.88	1.03
United States (US)	Thailand (TH)	219	0.94	1.05
China (CN)	Bulgaria (BG)	577	0.98	0.92
Barbados (BB)	Portugal (PT)	831	0.98	1.04
Korea (KR)	Netherlands (NL)	1,066	0.99	1.04
Georgia (GE)	Thailand (TH)	1,173	0.99	1.00
Bangladesh (BD)	India (IN)	4,381	1.00	1.05
Bangladesh (BD)	Netherlands (NL)	14,637	1.00	1.01
Iceland (IS)	Netherlands (NL)	65,918	1.00	0.78
Argentina (AR)	Thailand (TH)	131,851	1.00	1.01
Bangladesh (BD)	Georgia (GE)	131,851	1.00	0.97
Bangladesh (BD)	Germany (DE)	131,851	1.00	0.97
Bangladesh (BD)	Vietnam (VN)	131,851	1.00	0.96
Slovakia (SK)	Thailand (TH)	131,851	1.00	0.95
Iceland (IS)	Pakistan (PK)	131,851	1.00	0.96

### Selection pressure analyses

**Figure 9:**
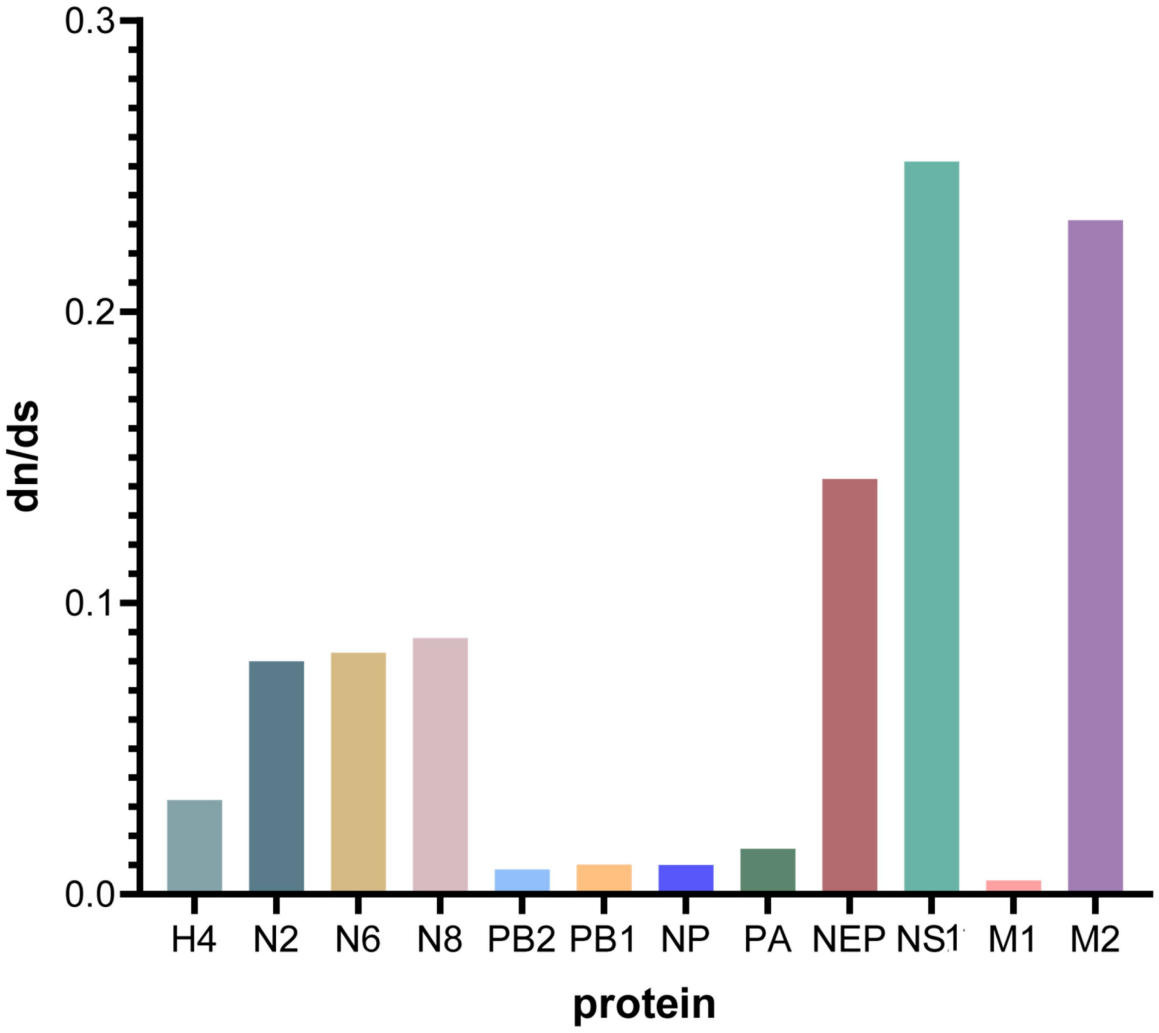
Comparative analysis of d*N*/d*S* ratios for each gene segment. Graphs were plotted using GraphPad Prism 10.0.0, with the *x*-axis indicating the protein of each gene fragment and the *y*-axis indicating the d*N*/d*S* value.

The sequences of the proteins encoded by H4N2, H4N6, and H4N8 were assessed for natural selection pressures, and the d*N*/d*S* ratios were calculated. The analysis showed that all d*N*/d*S* ratios were <1, indicating that H4, N2, N6, N8, PB2, PB1, NP, PA, M, and NS were under negative selection pressures (Table 4). The proteins encoded by the NS and M genes experienced high selection pressure, with NS1 and M2 proteins having the highest d*N*/d*S* ratios of 0.2517 and 0.2315, respectively. H4, N2, N6, and N8 experienced moderate selection pressures, and PB2, PB1, NP, and PA experienced the lowest selection pressures (Figure 9).

**Table 4:** Estimation of nonsynonymous and synonymous substitution rates (d*N*/d*S*) for each fragment of the H4NX AIV using Launch DnaSP6 software.

Gene	d*N*	d*S*	d*N*/d*S*
H4	0.0156	0.4802	0.0324
N2	0.0271	0.3384	0.0800
N6	0.0372	0.4484	0.0830
N8	0.0270	0.3062	0.0880
PB2	0.0040	0.4633	0.0085
PB1	0.0035	0.3430	0.0102
NP	0.0035	0.3471	0.0101
PA	0.0067	0.4249	0.0157
NEP	0.0484	0.3387	0.1427
NS1	0.0951	0.3778	0.2517
M1	0.0012	0.2396	0.0048
M2	0.0078	0.0338	0.2315

### Molecular characterization

To further analyze the potential threat of H4Nx AIVs to poultry, wild birds, and mammals, we performed molecular characterization of the 19 AIV strains (Table 5). The base cleavage sites of HA1 and HA2 of the HA genes of the 19 viral strains in this study were consistent with those of PEKASR/GLF, indicating that all viruses were of low pathogenicity. Receptor binding sites for all viral HA1 proteins showed Q226 and G228 (encoded by H3 Number), suggesting that the viruses are predisposed to avian-origin α-2,3-SA receptor binding. The NA genes of all viruses were present in the stems. The mutations E627K and D701N in PB2 imply enhanced viral replication in mammalian cells but were not found in any of the 19 virus strains in this study (Subbarao et al., 1993). However, all viruses were mutated at the L89V, G309D, and T339K sites of PB2, indicating enhanced polymerase activity and virulence in mammals (Zhang et al., 2023). It is concerning that the two strains, ZJ1686 and ZJ1674, have a mutation at the I292V site of PB2. This mutation is commonly found in human isolates and enhances the activity of the polymerase, resulting in high virulence and replication capacity of the virus in mice (Gao et al., 2019). Mutations in L473V of PB1, N30D and T215A of M1, and P42S and I106M of NS1 are associated with enhanced virulence in mice (Xu et al., 2012; Jiao et al., 2008; Kuo and Krug, 2009; Fan et al., 2009). Thus, all the viral strains in this study had mutation sites that enhanced virulence in mice, except for strains 352, 476, 289, DK2, and M50, which had alanine (A) at site 42 of the NS1 protein. The N66S mutation in the PB1-F2 protein was associated with increased viral pathogenicity and was present in all 19 viral strains in this study (Conenello et al., 2007). N383D and N409S mutations in PA, which enhance polymerase activity and mammalian fitness, were present in all viruses in this study (Yamayoshi et al., 2014). The S31N mutation in M2 indicates increased resistance of influenza viruses to amantadine (Wang et al., 2013), which was not present in the virus isolated in this experiment.

**Table 5:** Molecular labeling of viral gene segments.

Protein	Site	Virus																	
		177	74	60	352	ZJ1634	ZJ1586	ZJ1686	ZJ1534	ZJ1566	ZJ1498	ZJ1506	ZJ1674	ZJ476	ZJ1028	M50	DK2	ZJ1570	289
HA	Q226L	Q	Q	Q	Q	Q	Q	Q	Q	Q	Q	Q	Q	Q	Q	Q	Q	Q	Q
	G228S	G	G	G	G	G	G	G	G	G	G	G	G	G	G	G	G	G	G
PB2	E627K	E	E	E	E	E	E	E	E	E	E	E	E	E	E	E	E	E	E
	D701N	D	D	D	D	D	D	D	D	D	D	D	D	D	D	D	D	D	D
	I292V	I	I	I	I	I	I	V	I	I	I	I	V	I	I	I	I	I	I
	L89V	V	V	V	V	V	V	V	V	V	V	V	V	V	V	V	V	V	V
	G309D	D	D	D	D	D	D	D	D	D	D	D	D	D	D	D	D	D	D
	T339K	R	R	R	K	K	K	K	K	K	K	K	K	K	K	K	K	K	K
PB1	L473V	V	V	V	V	V	V	V	V	V	V	V	V	V	V	V	V	V	V
PB1-F2	N66S	S	S	S	S	S	S	N	S	S	S	S	N	S	S	S	S	S	S
PA	K356R	K	K	K	K	K	K	K	K	K	K	K	K	K	K	K	K	K	K
	N383D	D	D	D	D	D	D	D	D	D	D	D	D	D	D	D	D	D	D
	N409S	S	S	S	S	S	S	S	S	S	S	S	S	S	S	S	S	S	S
M1	N30D	D	D	D	D	D	D	D	D	D	D	D	D	D	D	D	D	D	D
	T215A	A	A	A	A	A	A	A	A	A	A	A	A	A	A	A	A	A	A
M2	S31N	S	S	S	S	S	S	S	S	S	S	S	S	S	S	S	S	S	S
NS1	P42S	S	S	S	A	S	S	S	S	S	S	S	S	A	S	A	A	S	A
	I106M	M	M	M	M	M	M	M	M	M	M	M	M	M	M	M	M	M	M

**Figure 10:**
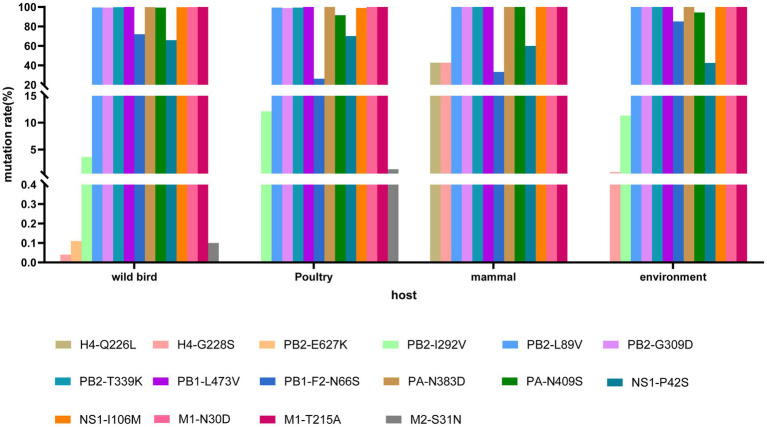
Mutation analysis of key amino acid sites in H4Nx AIV hosts. The associated amino acid changes were analyzed using MEGA 7.0. Consensus sequences were aligned, and mutations were recorded. The positions of the mutations for each enzootic cluster were confirmed manually. The number of amino acid changes in each enzootic cluster was counted.

To analyze amino acid substitutions in different host isolates of H4Nx viruses, nucleotide sequences from different hosts (wild birds, poultry, mammals, and the environment) were downloaded from the NCBI database; sequences with poor sequencing quality were deleted, and coding regions were retained after comparison. The mutation sites of each segment were quantified, and the 226 and 228 sites of the HA gene of H4Nx viruses were typically glutamine (Q) and glycine (G); however, H4Nx viruses isolated from mammals had a mutation rate of 42.86% in 226L and 228S. Moreover, mutations that may be associated with enhanced mammalian polymerase activity and mammalian virulence, such as PB2 (L89V, G309D, T339K), L473V of PB1, PA (N383D, N409S), I106M of NS1, and M1 (N30D, T215A) were found to be already prevalent in H4Nx viruses, and that these mutations have adapted to the evolution of the virus. The PB2-I292V locus has a 3.62% mutation rate in wild birds and a 12.12% mutation rate in poultry. The mutation rates of N66S in the PB1-F2 protein were 72.04, 26.37, 33.33, and 85.18% in wild birds, poultry, mammals, and the environment, respectively. The S31N mutation in the M1 protein, which implies increased viral resistance to amantadine, occurred sporadically only in wild birds and poultry, suggesting that H4Nx viruses are sensitive to amantadine (Figure 10).

## Discussion

Although AIVs of the H4Nx subtype are less pathogenic, they have a wide host range and have evolved to spread and recombine frequently among wild birds, poultry, and mammals; their internal genes can also serve as donors for highly pathogenic AIVs (Xu et al., 2023). Moreover, H4 AIVs are a common subtype of mixed infections found in live poultry markets (Luo et al., 2021), and a comprehensive analysis of H4 AIVs is imperative for human and poultry health.

H4Nx AIVs are globally prevalent, with the highest detection rates during 2006–2018, which may be attributed to the frequent invasion of HPAIV in Eurasia and North America since the discovery of HPAI H5N1 in 1996. This resulted in large numbers of deaths in poultry and a large number of deaths of wild birds infected with HPAIV in Qinghai Lake, China, in 2005, which led to increased surveillance efforts for wild birds and avian influenza in wild birds and poultry (Verhagen et al., 2021; Chen et al., 2006). Additionally, H4Nx AIV has been isolated primarily from wildfowl, with green-winged ducks being the most susceptible, followed by poultry (ducks), making waterfowl a natural host for H4Nx AIV (Webster et al., 1992). H4N6 viruses have replaced other subtypes as the major pandemic subtypes worldwide. Moreover, the effective population sizes of H4N2 and H4N8 viruses remained stable after a sharp decline after 2010 and that the effective population size of H4N6 showed a small increase in 2010, followed by a slow decline to remain stable, suggesting that the genetic diversity of H4N6 is greater than that of H4N4 and H4N8. According to the epidemiological survey of H4Nx AIV in China, it was primarily distributed in the southern part of the country, which is most likely because the southern region has a lot of waterfowl farming and is under the East Asia-Australasia migratory route, suggesting that the spread of H4Nx AIV may be related to the migration of wild birds.

Phylogenetic analysis showed that the H4Nx AIV internal genes in this study clustered in the same branch with high homology to the internal genes of the highly pathogenic H5N1 and H5N8, further confirming that H4Nx AIV may provide internal genes for HPAIV. Concurrently, the M gene of ZJ1634 (H4N6) and ZJ1566 (H4N6) strains was identified as being closely related to that of the A/swine/Guangdong/K4/2011 (H4N8) strain, sharing a 96% homology as determined by NCBI’s analysis of the AIVs sequenced in our study. Swine are often referred to as a “mixing vessel” for influenza viruses due to their propensity for viral reassortment among avian, human, and porcine populations. A noteworthy study reported that the isolation of H4N8 avian influenza virus from pigs in Guangdong is the first report of cross-species transmission of avian H4N8 influenza virus to domestic pigs under natural conditions (Su et al., 2012). This finding suggests that the potential for avian-origin H4N8 AIV can be transmitted across species and infect swine populations.

The branch where the surface genes of the virus in this study are located is clustered with numerous viruses of poultry (duck) origin. This suggests that the subpopulation where the viruses isolated in this study are located has been frequently introduced from wild birds to poultry. Moreover, these viruses are closely related to H4N6 AIV isolated from wild birds, poultry, and the environment from Korea, Mongolia, Vietnam, and Japan. It has been reported that the N8 gene, which is derived from H4N8 in China, appears in the North American spectrum, suggesting that the North American N8 gene has been introduced into Eurasia (Lin et al., 2024). The two N8 strains isolated in this study are consistent with this characterization. H4Nx Avian influenza A viruses act as gene donors and can undergo complex recombination with other subtypes of H1, H3, H5, H6, and H7 to produce new recombinant viruses (Teng et al., 2012). It has been shown that wild birds can carry low-pathogenic AIVs that can infect poultry and mammals and have a strong capacity for interspecies transmission (Kim et al., 2021). In summary, H4Nx avian influenza A viruses may be transmitted to mammals and poultry through wild birds.

Additionally, this study showed that H4Nx AIVs circulate in Europe and Asia, with the Netherlands and Bangladesh having the most intensive migratory routes. This finding suggests that the virus spreads most intensively and frequently in these two countries, indicating a high prevalence of H4 AIVs encompassing a rich variety of subtypes in Europe and Asia. Moreover, the migratory movements of wild birds are inextricably linked to the spread of AIV (Jeong et al., 2019; van der Kolk, 2019). For example, the Netherlands is an important wintering ground for wild birds, and large numbers of wild birds migrate to the Netherlands, where an average of 1.5 million mallards winter each year (van der Kolk, 2019), providing conditions for the spread of LPAIV. Furthermore, Bangladesh has a high prevalence of AIV in poultry, substandard biosecurity in live poultry markets, and frequent movement of live poultry markets to and from poultry farms, which facilitates the spread of AIV; therefore, poultry are considered the primary host for AIV in this region (Hassan et al., 2017; Hassan et al., 2018). Foreign sales of poultry and poultry products may accelerate the spread of H4Nx AIVs.

The receptor binding sites of the 19 strains of the virus in this study were 226Q and 228G, which preferentially bind to the avian receptor and were found to be prevalent in poultry and wild birds, which were more susceptible to infection by H4Nx AIV. However, the receptor binding sites of the virus isolated from swine were 226L and 228S, which were altered to be more susceptible to binding to the human receptor. The related mutations, E627K and D701N, which enable enhanced mammalian replication and virulence, were not found in the viruses in this study. The PB2-I292V mutation, commonly observed in human pandemic H1N1 AIV, is a potential mammalian adaptive mutation that contributes to viral adaptation and survival in mammals (Gao et al., 2019). Only two of the 19 strains of the virus in this study were mutated in I292V of PB2, which was found to be more prevalent in poultry than in wild birds. The N66S mutation in the PB1-F2 protein enhances viral virulence by inhibiting the interference response (Conenello et al., 2011), and this mutation may have contributed to the high lethality of the H5N1 AIV in 1918 (Conenello et al., 2007). In this study, 17 viral strains were mutated at PB1-F2-N66S, and the highest mutation rate was observed in wild birds. Other mutations associated with enhanced polymerase activity and enhanced mammalian virulence, such as PB2 (L89V, G309D, and T339K), PB1-L473V, M1 (N30D and T215A), and PA (N383 and N409S), are prevalent in H4Nx AIV, suggesting that H4Nx AIV are potentially threatening to humans and mammals.

The ratio of nonsynonymous substitutions (d*N*) to synonymous substitutions (d*S*) is commonly used to determine the type of natural selection pressure that occurs in protein-coding sequences. Adaptation of the virus to a new host is one of the primary factors driving the evolution of AIV, and when selection pressure is greater, it indicates that the adaptive evolution of the virus has occurred (Han et al., 2019). In this study, the virus was under negative selection pressure; however, the NS1 and M2 proteins had higher d*N*/d*S* ratios, suggesting that these genes were under stronger selection pressure and were more susceptible to adaptive evolution.

Wild birds are the primary hosts of H4Nx viruses, and host species have been shown to correlate with viral recombination; wild birds are the primary source of newly mutated strains of the virus (Lu et al., 2014). Therefore, continuous monitoring of AIVs in wild birds is crucial. In summary, our findings provide a basis for understanding the evolution and transmission pathways of H4Nx AIVs, and for the prevention and control AIVs.

## Data Availability

The original contributions presented in the study are included in the article/Supplementary material, further inquiries can be directed to the corresponding authors.
